# Mutations in Protocadherin 15 and Cadherin 23 Affect Tip Links and Mechanotransduction in Mammalian Sensory Hair Cells

**DOI:** 10.1371/journal.pone.0019183

**Published:** 2011-04-21

**Authors:** Kumar N. Alagramam, Richard J. Goodyear, Ruishuang Geng, David N. Furness, Alexander F. J. van Aken, Walter Marcotti, Corné J. Kros, Guy P. Richardson

**Affiliations:** 1 Otolaryngology Head and Neck Surgery, University Hospitals Case Medical Center, Case Western Reserve University, Cleveland, Ohio, United States of America; 2 School of Life Sciences, University of Sussex, Falmer, Brighton, United Kingdom; 3 Institute for Science and Technology in Medicine, School of Life Sciences, Keele University, Staffordshire, United Kingdom; 4 Department of Biomedical Science, University of Sheffield, Sheffield, United Kingdom; Claremont Colleges, United States of America

## Abstract

Immunocytochemical studies have shown that protocadherin-15 (PCDH15) and cadherin-23 (CDH23) are associated with tip links, structures thought to gate the mechanotransducer channels of hair cells in the sensory epithelia of the inner ear. The present report describes functional and structural analyses of hair cells from *Pcdh15^av3J^* (*av3J*), *Pcdh15^av6J^* (*av6J*) and *Cdh23^v2J^* (*v2J*) mice. The *av3J* and *v2J* mice carry point mutations that are predicted to introduce premature stop codons in the transcripts for *Pcdh15* and *Cdh23*, respectively, and *av6J* mice have an in-frame deletion predicted to remove most of the 9th cadherin ectodomain from PCDH15. Severe disruption of hair-bundle morphology is observed throughout the early-postnatal cochlea in *av3J/av3J* and *v2J/v2J* mice. In contrast, only mild-to-moderate bundle disruption is evident in the *av6J/av6J* mice. Hair cells from *av3J/av3J* mice are unaffected by aminoglycosides and fail to load with [^3^H]-gentamicin or FM1-43, compounds that permeate the hair cell's mechanotransducer channels. In contrast, hair cells from *av6J/av6J* mice load with both FM1-43 and [^3^H]-gentamicin, and are aminoglycoside sensitive. Transducer currents can be recorded from hair cells of all three mutants but are reduced in amplitude in all mutants and have abnormal directional sensitivity in the *av3J/av3J* and *v2J/v2J* mutants. Scanning electron microscopy of early postnatal cochlear hair cells reveals tip-link like links in *av6J*/*av6J* mice, substantially reduced numbers of links in the *av3J/av3J* mice and virtually none in the *v2J/v2J* mice. Analysis of mature vestibular hair bundles reveals an absence of tip links in the *av3J*/*av3J* and *v2J*/*v2J* mice and a reduction in *av6J*/*av6J* mice. These results therefore provide genetic evidence consistent with PCDH15 and CDH23 being part of the tip-link complex and necessary for normal mechanotransduction.

## Introduction

Mouse mutants have played an important role in the identification of genes linked to hereditary hearing loss and provide *in vivo* models for investigating gene function (see [1], [2], [3], [4] for recent reviews). Many of the deaf mouse mutants have defects in the sensory hair bundle, the structure that detects the sound-induced movements of the cochlear fluids and transduces these stimuli into electrical signals. The hair bundle is located at the apical end of the hair cell and is composed of two or more height-ranked rows of stereocilia that are coupled to one another by several types of link. All hair bundles, apart from those in the mature auditory organs of mammals, also have a single kinocilium that lies adjacent to, and is linked to, the tallest row of stereocilia. The tip link, an obliquely directed filament that connects the tip of a stereocilium to the side of a neighbouring stereocilium, is thought to gate the hair cell's mechanotransducer channel [5], [6]. Although not yet identified, this channel is now thought to be located around the tips of all but the tallest stereocilia, near the basal or lower end of the tip link in cochlear hair cells [7].

Considerable evidence now indicates that the tip link is composed of two proteins, protocadherin 15 (PCDH15) and cadherin 23 (CDH23). High resolution images [8] and Fourier analysis [9] reveal that tip links consist of two intertwined strands, and immunocytochemical studies have shown PCDH15 and CDH23 are both associated with tip links, with PCDH15 localising to the lower end of the tip link and CDH23 to the upper end [10], [11], [12], [13]. A widely accepted model [14] for the tip links proposes that PCDH15 and CDH23 form cis-homodimers that interact in trans via their opposing N-termini [15], [16] to form a tip link that is ∼175 nm long, as predicted by the combined lengths of the repeats in the ectodomains of CDH23 and PCDH15 (27 and 11 cadherin repeats, respectively). Functional evidence for such a model is less abundant, although the zebrafish sputnik mutant reveals that cadherin 23 is required for tip link formation [17] and the salsa mouse with a missense mutation in the ectodomain of *Cdh23* shows age-related hearing loss that correlates with a loss of tip links [18]. Also, recombinant fragments encompassing the putative interaction domains of PCDH15 and CDH23 block the development and regeneration of mechanotransduction in hair cells in vitro [19]. In this study we examined mechanotransduction and tip links in three mouse mutants, the *av3J* [20] and *av6J* [21] Ames waltzer mice which have effective null and missense mutations, respectively, in *Pcdh15*, and the *v2J* waltzer mouse with a splice-site mutation in *Cdh23* [22]. The results provide further functional and genetic evidence supporting the current model for tip link structure and function.

## Results

**Figure 1:**
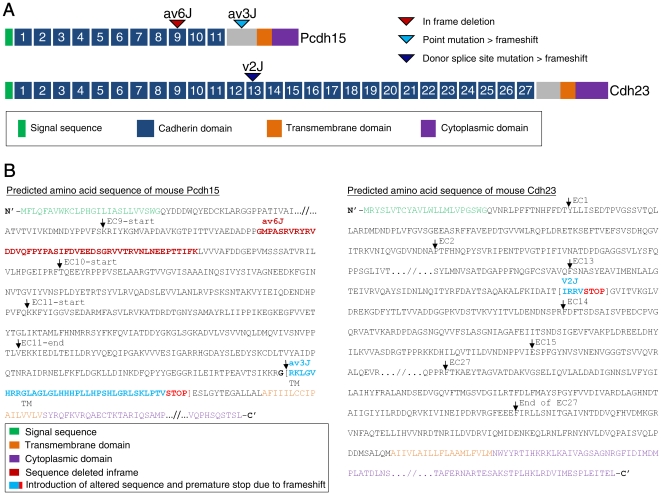
Mutations in *av6J*, *av3J* and *v2J* mice. (A) Schematic representation of the mouse PCDH15 and CDH23 proteins. The location of the *av6J* and *av3J* mutations in *Pcdh15*, and the *v2J* mutation in *Cdh23* are indicated. (B) Changes in the amino acid sequences of PCDH15 and CDH23 resulting from the mutations.

The present report focuses on functional and structural analysis of hair cells from *Pcdh15^av3J^* (*av3J*), *Pcdh15^av6J^* (*av6J*) and *Cdh23^v2J^* (*v2J*) mice. The *av3J* mice carry a presumptive null allele of *Pcdh15*, and the *av6J* mice carry an in-frame deletion predicted to remove most of the 9^th^ cadherin repeat from the ectodomain of PCDH15 (Fig. 1A). The *v2J* mice have a splice donor site mutation (Fig. 1A) predicted to result in exon skipping and premature truncation of the open reading frame [22]. Changes to the amino acid sequence for all three alleles are shown in Figure 1B.

### Cochlear hair bundle structure in early postnatal mutants

**Figure 2:**
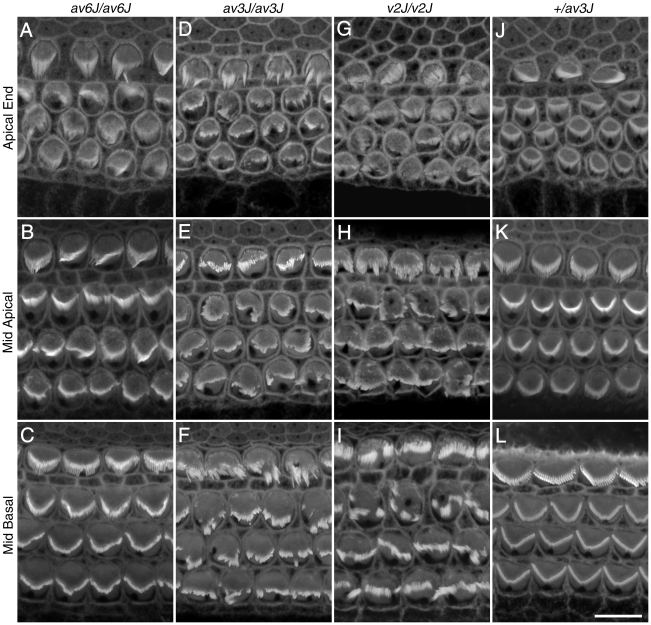
Bundle morphology in phalloidin stained *av6J*, *av3J* and *v2J* cochlear hair cells at P3. Images are from homozygous *av6J/av6J* (A–C), *av3J/av3J* (D–F), *v2J/v2J* (G–I) mice and a heterozygous *+/av3J* control (J–L) mouse. Mild to moderate disruption of the hair bundles was observed in the cochlea of *av6J/av6J* mice (A–C). Severe damage to all regions was observed in both the *av3J/av3J* (D–F) and the *v2J/v2J* (G–I) mutants. Scale bar = 10 µm.

The functional analysis of the *av6J*, *av3J* and *v2J* mutants described in this paper was performed on cochlear cultures and acute preparations derived from the early postnatal cochlea because the hair cells are known to be particularly suitable for studying mechanotransduction at this stage of development [23], [24]. Figure 2 compares confocal images of phalloidin-stained cochlear hair bundles from *av6J*/*av6J* (Fig. 2A–C), *av3J/av3J* (Fig. 2D–F), *v2J/v2J* (Fig. 2G–I) and heterozygous *+/av3J* (Fig. 2J–L) mice at P3. The hair bundles of the *av6J/av6J* mutant (Fig. 2A–C) showed the least signs of disruption, whilst those of the *v2J/v2J* mouse (Fig. 2G–I) were the most severely disorganised. In all three mutants, hair-bundle structure was disrupted throughout the length of the cochlea (Fig. 2A–I).

### Reduced *Pcdh15* ectodomain staining in *av6J/av6J* mutants

Previously published immunocytochemical data have shown that the *av3J/av3J* and *v2J/v2J* mice are effective nulls for *Pcdh15* and *Cdh23* respectively [11], [25]. To determine whether PCDH15 is expressed in the hair cells of the *av6J/av6J* mutant, whole-mount preparations of P3 cochleae from *+/av6J* and *av6J/av6J* mice were stained with antibodies directed against the intracellular domain of the three major transmembrane forms of PCDH15 that are known to be expressed in the mouse ear, CD1, CD2 and CD3 [11]. Cochlear cultures were also stained with HL5614, an antibody that was raised to a large recombinant fragment encompassing the first two cadherin repeats of the ectodomain of PCDH15, a region that is common to many of the 24 different *Pcdh15* mRNA isoforms that have been detected by RT-PCR in the early postnatal cochlea [11]. All these antibodies have been previously validated [11].

**Figure 3:**
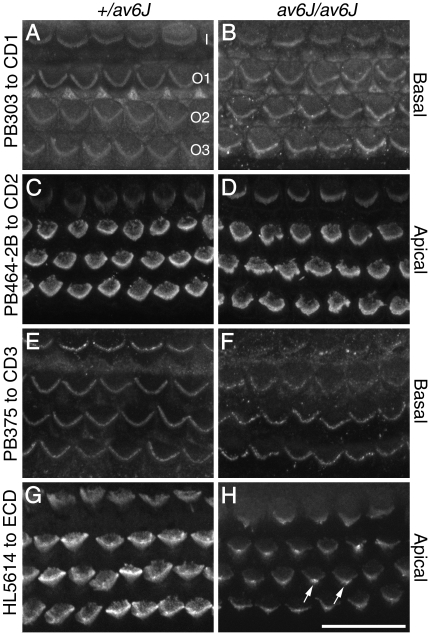
Distributions of PCDH15-CD1, -CD2, -CD3 and -ectodomain (ECD) antigen HL5614 in the organ of Corti of *av6J* mice at P3. Hair bundles from *+/av6J* (A, C, E, G) and *av6J/av6J* (B, D, F, H) mice were stained with antibody PB303 to PCDH15-CD1 (A, B), antibody PB464-2B to PCDH15-CD2 (C, D), antibody PB375 to PCDH15-CD3 (E, F) and antibody HL5614 to PCDH15-ectodmain (G, H). Images are from the basal (A, B, E, F) and apical (C, D, G, H) coils. Staining seen with antibodies to the CD1 (A, B), CD2 (C, D) and CD3 (E, F) isoforms of PCDH15 is similar in *+/av6J* (A, C, E) and *av6J/av6J* (B, D, F) mice. Staining seen with HL5614 to the PCDH15 ectodomain in *av6J/av6J* (H) hair bundles is weak in comparison to that seen in *+/av6J* hair bundles (G) and is most prominent in the region of the kinocilium (arrows). I, Inner hair cell; O1, O2, O3, outer hair cells in rows 1, 2, and 3 respectively. Scale bar = 20 µm.

At P3, the antibodies to CD1 and CD3 stain the hair bundles of basal-coil hair cells more intensely than those at the apex of the cochlea, whilst antibodies to CD2 and the ectodomain show the reverse pattern, staining hair bundles in the apex more intensely than those in the base. At any given point along the cochlea, the hair-bundle staining observed in the *+/av6J* and *av6J/av6J* mice with antibodies to CD1 (Fig. 3A, B), CD2 (Fig. 3C, D) and CD3 (Fig. 3E, F) is similar, both in terms of intensity and pattern. Anti-CD1 and anti-CD2 label the entire bundle and anti-CD3 labels punctae at the tips of stereocilia (Fig. 3 A–F). In contrast, the intensity of hair-bundle staining observed with the ectodomain antibody HL5614 was much reduced in the *av6J/av6J* mutant relative to that observed in the *+/av6J* heterozygote, and concentrated on the stereocilia lying in proximity to the kinocilium (Fig. 3G–H). These results suggest that the *av6J* mutation may lead to degradation or loss of the ectodomain from the transmembrane variants, or specifically reduce the expression of the 4^th^ isoform class that is predicted to lack the transmembrane and intracellular domains and may be secreted.

### FM1-43 loading is reduced in av6J/av6J mutants and abolished in *av3J/av3J* & *v2J/v2J* mutants

**Figure 4:**
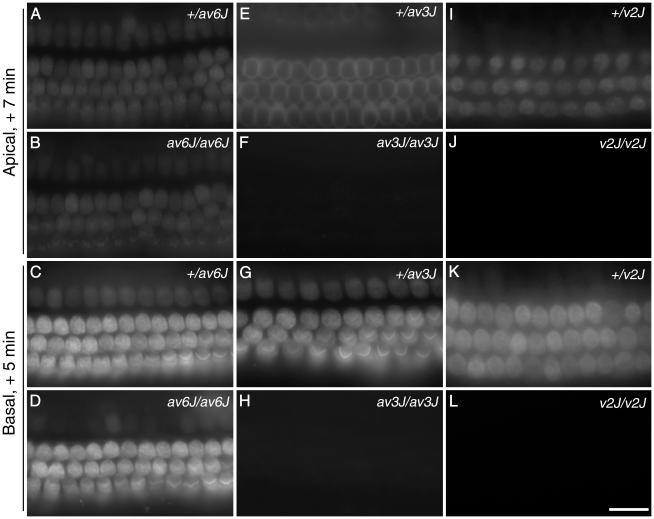
FM1-43 uptake in *av6J*, *av3J* and *v2J* cochleae. Images are from *+/av6J* (A, C), *av6J/av6J* (B, D), *+/av3J* (E, G), *av3J/av3J* (F, H), *+/v2J* (I, K) and *v2J/v2J* (J, L) cochlear hair cells at the equivalent of P3. In the apical turn and basal coils of the *av6J/av6J* mouse (B, D) uptake is diminished compared to the control (A, C). In contrast to hair cells from *av6J/av6J* mice, hair cells from *av3J/av3J* (F, H) and *v2J/v2J* (J, L) mice completely fail to load with FM1-43 in both apical (F, J) and basal (H, L) coils. Uptake in heterozygous *+/av6J* (A, C), *+/av3J* (E, G) and *+/v2J* mice (I, K) is comparable. Scale bar = 20 µm.

FM1-43 is an amphipathic styryl dye that is known to rapidly accumulate in sensory hair cells via the mechanotransducer channels that are open at rest in non-stimulated hair bundles [26], [27]. Cochlear cultures from *av6J* (Fig. 4A–D), *av3J* (Fig. 4E–H) and *v2J* (Fig. 4I–L) mice were examined for their ability to load with FM1-43 in response to a brief, 10 second exposure to the dye. Hair cells from *av6J/av6J* mice showed dye loading both in the apical and the basal turns of the cochlea (Fig. 4B, D). Quantitative analysis indicated a 20–25% reduction in dye loading in both the apical and basal coils of the *av6J/av6J* mutants relative to that observed in the heterozygous *+/av6J* controls. In the apical coils the mean grey scale levels were 45.9±3.0 (n = 11) in the *+/av6J* heterozygotes versus 34.9±2.8 (n = 6) in the *av6J/av6J* mutants, and in the basal coils the mean grey scale levels were 87.4±4.6 (n = 11) in the *+/av6J* heterozygotes versus 69.6±7.1 (n = 6) in the *av6J/av6J* mutants. The differences in grey scale levels between heterozygotes and mutants were not statistically significant (ANOVA plus Tukey test). In contrast, loading of FM1-43 dye was not detected at all in hair cells from either the apical or the basal coils of cochlear cultures prepared from *av3J/av3J* (Fig. 4F, H) and *v2J/v2J* mice (Fig. 4J, L). These results indicate that hair cells from *av6J/av6J* mice, but not those from *av3J/av3J* or *v2J/v2J* mice, have mechanotransducer channels that are open at rest.

### Gentamicin loading and aminoglycoside sensitivity in Pcdh15 mutants

**Figure 5:**
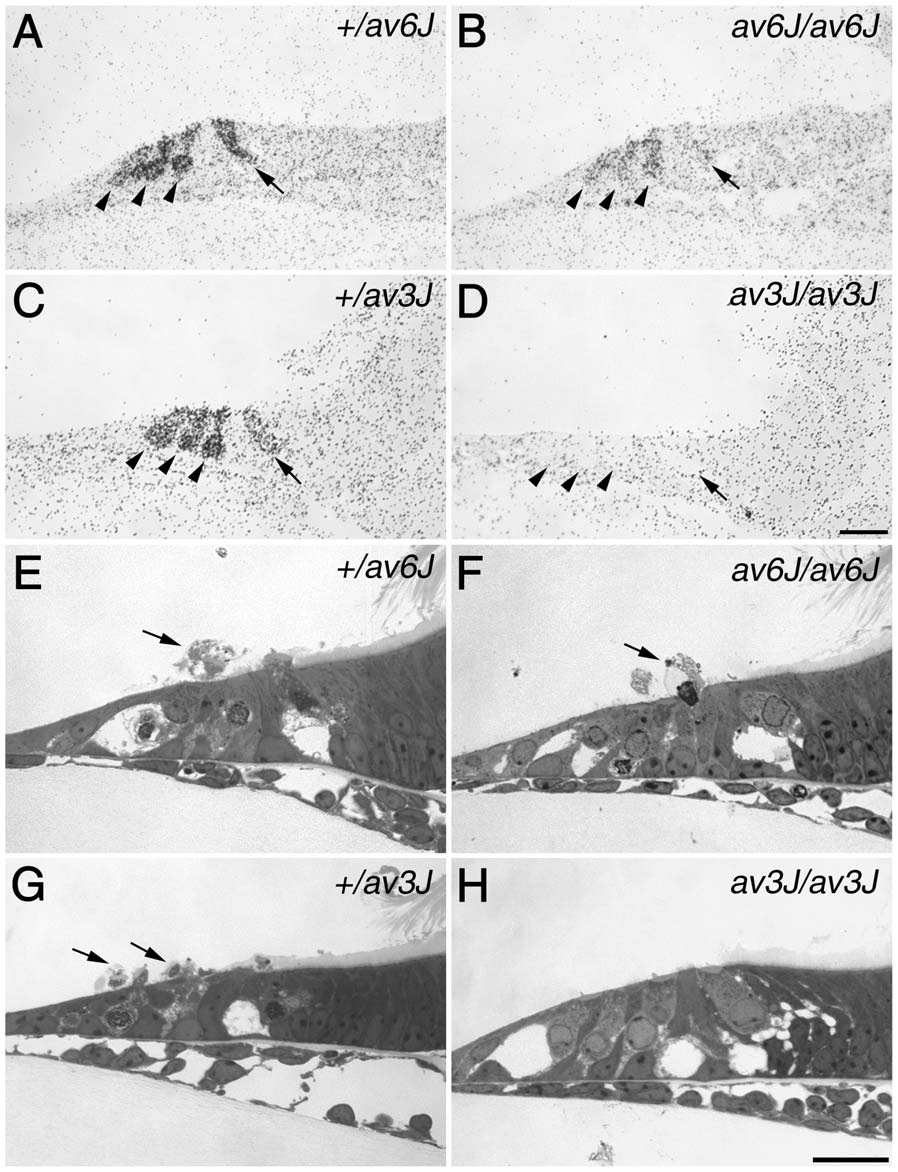
[^3^H]-gentamicin labelling and gentamicin toxicity in *av6J* and *av3J* cochlear cultures. (A–D) Autoradiographs of [^3^H]-gentamicin uptake in *+/av6J* (A), *av6J/av6J* (B), *+/av3J* (C) and *av3J/av3J* (D) apical-coil hair cells. Arrowheads indicate outer hair cells, arrows indicate inner hair cells. [^3^H]-gentamicin uptake observed in the *av6J/av6J* mouse (B) is reduced relative to that in the *+/av6J* control (A). In contrast, *av3J/av3J* hair cells (D) completely fail to load with [^3^H]-gentamicin whilst *+/av3J* control loading (C) is similar to that observed for *+/av6J* hair cells (A). (E–H) Toluidine blue stained light micrographs of gentamicin treated cochlear cultures from *+/av6J* (E), *av6J/av6J* (F), *+/av3J* (G) and *av3J/av3J* (H) mice. Arrows indicate extruded hair cells. Whilst *+/av6J* (E), *av6J/av6J* (F) and *+/av3J* hair cells (G) are all sensitive to gentamicin, *av3J/av3J* hair cells (H) are resistant to this antibiotic. Scale bars = 20 µm.

Hair cells in cochlear cultures prepared from shaker1 mouse mutants that have a mutation in myosin VIIa and have all transducer channels closed at rest [26], [28], fail to load with FM1-43 [26], do not load with [^3^H]-gentamicin and are resistant to aminoglycoside-induced ototoxicity [29]. Furthermore, there is evidence that the aminoglycoside antibiotics are, like FM1-43, permeant blockers of the hair cell's mechanotransducer channels [30]. [^3^H]-gentamicin accumulation and sensitivity to aminoglycoside antibiotics were therefore examined and compared in the *av6J/av6J* and *av3J/av3J* mutants. Following exposure to 0.1 mM [^3^H]-gentamicin for a period of 2 h at 37°C, the selective accumulation of radioactive label was observed in the inner and outer hair cells of the *av6J/av6J* mutant but reduced relative to that seen in the heterozygous *+/av6J* control (Fig. 5A, B). Labelling was not observed in the hair cells of the *av3J/av3J* mutant relative to that observed in the heterozygous *+/av3J* control (Fig. 5C, D). As expected from these data, the hair cells in the *av6J/av6J* mutant were damaged and extruded from the epithelium when exposed to 1 mM gentamicin for 3 h at 37°C (Fig. 5E, F), whilst those of the *av3J/av3J* mutant were not (Fig. 5G, H).

### Transducer currents are abnormal in *av6J/av6J*, *av3J/av3J* and *v2J/v2J* mutant outer hair cells

**Figure 6:**
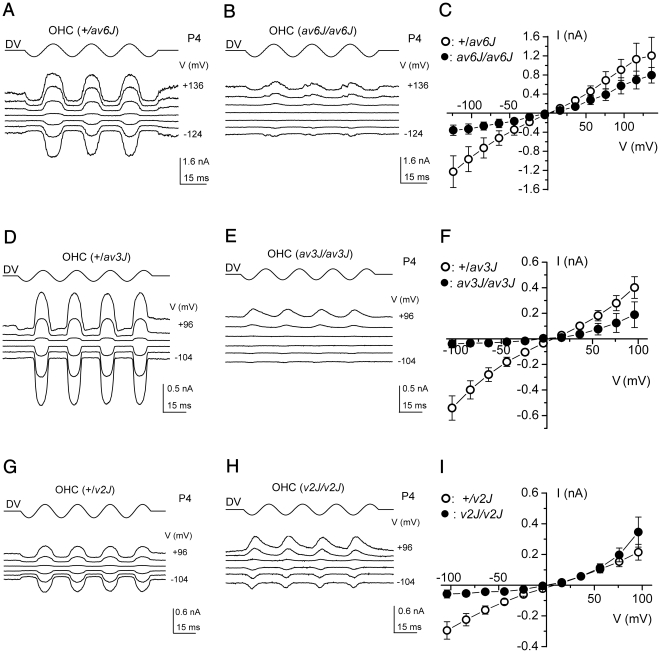
Mechano-electrical transduction in *av6J*, *av3J* and *v2J* outer hair cells. (A, B) Transducer currents from heterozygous *+/av6J* and homozygous *av6J/av6J* P4 OHCs in response to 45 Hz sinusoidal force stimuli. Holding potential was −84 mV and the membrane potential was stepped, in 20 mV increments, between −124 mV and +136 mV. For clarity only responses to every third voltage step are shown. Driver voltage (DV, amplitude 40 V) to the fluid jet is shown above the traces. Positive DVs move the hair bundle towards the kinocilium. Membrane potentials are shown next to some of the traces. Recordings shown are single traces and are offset so that the zero-transducer current levels are equally spaced. (A): C_m_ 2.9 pF; R_s_ 1.9 MΩ. (B): C_m_ 3.4 pF; R_s_ 1.4 MΩ. (C) Averaged current-voltage curves, measured peak-to-peak from two *+/av6J* OHCs (one P4 and one P5) and three *av6J/av6J* OHCs (one P4 and two P5). (D, E) Transducer currents from heterozygous *+/av3J* and homozygous *av3J/av3J* P4 OHCs. Cells were held at −84 mV and the membrane potential was stepped, in 20 mV increments, between −104 mV and +96 mV. Only responses to every other voltage step are shown. Driver voltage was 35 V. Recordings in (D) and (E) are averages of 4 repetitions each, (D): C_m_ 6.2 pF; R_s_ 1.9 MΩ. (E): C_m_ 5.8 pF; R_s_ 1.5 MΩ. (F) Average current-voltage curves, measured peak-to-peak, for five *+/av3J* (P2+2) and five *av3J/av3J* (P2+2) OHCs. (G, H) Transducer currents from heterozygous *+/v2J* (P0+3) and homozygous *v2J/v2J* (P1+2) OHCs, stimulus protocol and data presentation as in (D, E). Recordings averaged from 3 (G) and 2 (H) repetitions. (G): C_m_ 5.9 pF; R_s_ 4.3 MΩ. (H): C_m_ 4.9 pF; R_s_ 4.0 MΩ. (I) Averaged current-voltage curves, measured peak-to-peak from two *+/v2J* OHCs (P0+3) and three *v2J/v2J* (P0+3 and P1+2) OHCs.

To determine whether the mechanotransducer channels could be activated by hair bundle displacements in the mutants, currents were measured in the whole cell mode whilst stimulating the hair bundles with a fluid jet (Fig. 6A–I). Heterozygous *+/av6J*, *+/av3J* and *+/v2J* outer hair cells (OHCs) all responded with large transducer currents when the hair bundles were deflected in the positive direction (towards the kinocilium) using saturating force stimuli (Fig. 6A, D, G). At −104 mV the size of the transducer currents was −970 pA for the *+/av6J* OHCs (range −700 to −1230 pA), −540 pA for the *+/av3J* OHCs (range −370 to −850 pA) and −300 pA for the *+/v2J* OHCs (range −240 to −350 pA), showing considerable variability as reported before for rat OHCs [31]. Such variability may reflect differences between OHCs in the numbers of intact tip-link – transducer channel complexes surviving experimental procedures, a variable that may also differ between the strains of mice used. When the bundles were moved in the negative direction (away from the kinocilium), any transducer channels open in the absence of mechanical stimulation (normally about 5–10%) were closed, resulting in a reduction of the inward current. Starting from −104 mV and stepping the membrane potential to more depolarized values, the transducer currents decreased in size at first and then reversed at close to 0 mV. For more positive membrane potentials, fluid jet stimuli in the positive direction caused outward currents and the fraction of transducer channels open without stimulation increased, probably as a consequence of reduced adaptation due to a decreased driving force for Ca^2+^ ions through the transducer channels [32].

In the *av6J/av6J* mutants, transducer currents could be elicited over the range of membrane potentials tested (Fig. 6B). The current-voltage curves (Fig. 6C), averaged for two *+/av6J* and three *av6J/av6J* OHCs, show that the amplitudes were smaller in the homozygotes by about 65% at −104 mV, gradually reducing to 37% at +96 mV. Also, a small resting transducer current was evident at negative potentials that increased at positive potentials. When the same voltage step protocol was applied to homozygous *av3J/av3J* OHCs the transducer current was absent or small at all but the most positive potentials (Fig. 6E), where clear current responses could be seen. These currents were, surprisingly, in response to the negative phase of the sinusoidal force stimulus that would move the bundle away from the kinocilium. Figure 6F shows the averaged current-voltage curves for the peak-to-peak transducer currents of five *+/av3J* and five *av3J/av3J* OHCs. The amplitudes of the transducer currents were considerably smaller in the homozygous mutant OHCs than those measured in the heterozygous controls, by 93% at −104 mV. The reduction in current decreased gradually at positive membrane potentials, down to 53% at +96 mV. One of the five *av3J/av3J* OHCs had, when stimulated with larger sinusoidal force stimuli (45 V driver voltage amplitude), larger transducer currents in response to stimuli directed away from the kinocilium, also at negative membrane potentials. This suggests that large transducer currents can be elicited, but they are not activated at rest and large forces are required to evoke them. The homozygous *v2J/v2J* mutants had transducer currents at all membrane potentials (Fig. 6H), but again mostly in response to force directed away from the kinocilium. Currents at negative membrane potentials were smaller than those at positive membrane potentials and reached a peak earlier, suggestive of strong adaptation. No resting transducer current occurred at any potential. The current-voltage curves (averaged from two *+/v2J* and three *v2J/v2J* OHCs) show the relatively small transducer currents observed at negative potentials for the homozygous mutant OHCs, which were 81% smaller than those of the heterozygous controls at −104 mV. Note that at positive potentials the currents are actually larger in the homozygotes, most likely because the heterozygous control currents happened to be rather small in this sample of two OHCs from a single mouse.

**Figure 7:**
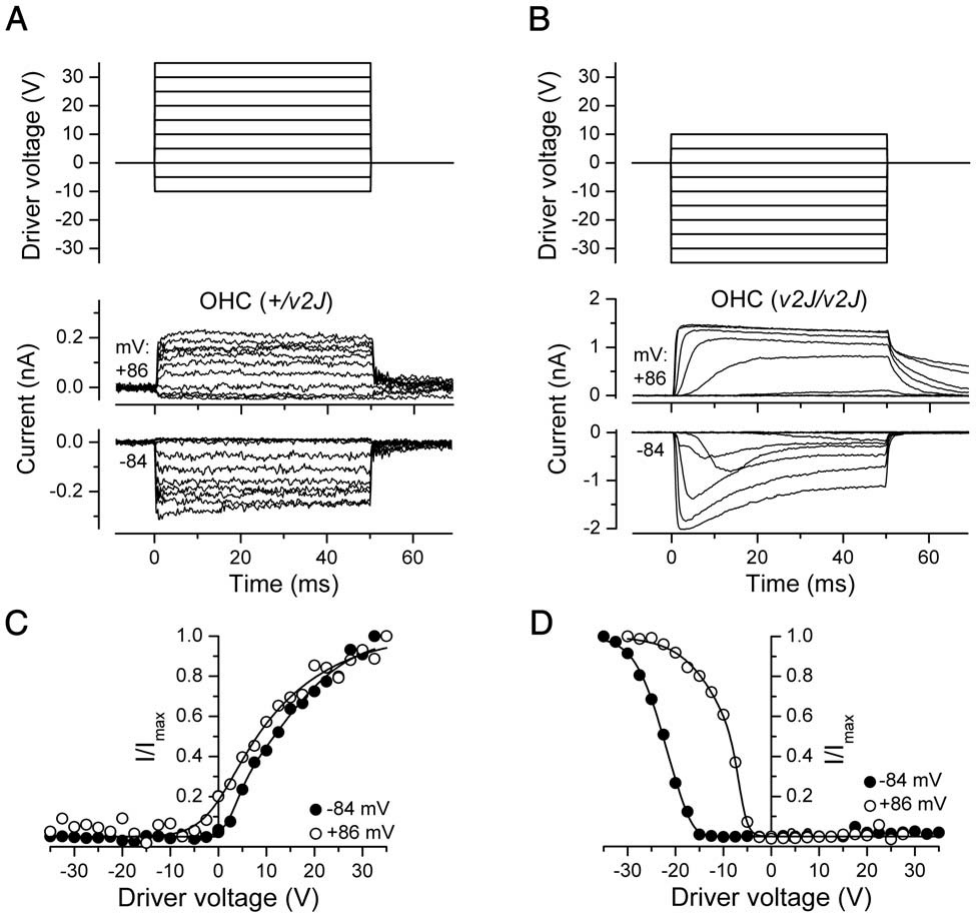
Mechanoelectrical transduction of *v2J* outer hair cells in response to force steps. (A, B) Transducer currents recorded at membrane potentials of −84 mV and +86 mV from a *+/v2J* (A) and a *v2J/v2J* (B) OHC. The *+/v2J* OHC (same cell as Fig. 6G) responds with transducer currents (inward at −84 mV, outward at +84 mV) to force steps in the positive direction (elicited by positive driver voltages to the fluid jet as shown above the current traces). Force steps in the negative direction (elicited by negative driver voltages) shut off the small fraction of transducer channels open at rest. Transducer currents of the *v2J/v2J* OHC are elicited by force steps in the negative direction and adapt strongly at −84 mV but not at +86 mV. There is no transducer current activated at rest. (C, D) Peak transducer current as a function of driver voltage to the fluid jet. Smooth curves are 2nd order Boltzmann functions: I = I_max_/(1+exp(a_2_(DV_2_-DV))*(1+exp(a_1_(DV_1_-DV)))). Fits for *+/v2J* are at −84 mV, a_1_ = 0.617 V^−1^, a_2_ = 0.127 V^−1^, DV_1_ = 3.42 V, DV_2_ = 11.5 V, and at +86 mV, a_1_ = 0.230 V^−1^, a_2_ = 0.095 V^−1^, DV_1_ = 2.15 V, DV_2_ = 5.67 V. Fits for *v2J/v2J* are at −84 mV, a_1_ = −0.663 V^−1^, a_2_ = −0.318 V^−1^, DV_1_ = −16.7 V, DV_2_ = −22.6 V, and at +86 mV, a_1_ = −0.972 V^−1^, a_2_ = −0.196 V^−1^, DV_1_ = −7.00 V, DV_2_ = −7.69 V.

One concern about the unusual electrical responses of the *av3J/av3J* and *v2J/v2J* OHCs is that they might not be transducer currents but some artefact, for example, of modulating the seal resistance between the patch pipette and the cell membrane. The currents in one of these mutants, *v2J/v2J*, were therefore investigated further, using mechanical step stimuli. At a hyperpolarized holding potential of −84 mV a considerably larger driver voltage to the fluid jet, and hence force on the hair bundle, was needed to elicit currents from the mutant *v2J/v2J* OHC than was required for the control *+/v2J* OHC (Fig. 7). Whereas the control cell responded maximally to force stimuli in the positive direction (Fig. 7A, C), the mutant OHC responded best to negatively directed stimuli (Fig. 7B, D). Of the five mutant OHCs tested using force steps, one responded exclusively to positive, one exclusively to negative, and three mainly to negative steps, but with smaller responses also to positive steps. The currents in all five mutant OHC showed strong adaptation at −84 mV during the force steps (example in Fig. 7B), and considerably more so than in the heterozygous controls (Fig. 7A). Adaptation was no longer evident when the membrane potential was stepped to +86 mV (Fig. 7A, B), at which potential the currents in the mutant OHCs decayed quite slowly upon termination of the force step (Fig. 7B). Currents also developed more slowly in the mutant OHCs, with onset kinetics speeding up with the larger steps (Fig. 7B). In three mutant OHCs, bundle displacements were measured and it was found that a bundle displacement of 57±16 nm (in the negative direction) was required to elicit a transducer current at −84 mV. At a depolarized membrane potential of +86 mV the relation between currents and driver voltage shifted towards smaller driver voltages for both the control *+/v2J* OHCs and the mutant *v2J/v2J* OHCs (Fig. 7C, D), an observation usually interpreted as being due to abolished Ca^2+^-induced adaptation upon approaching the Ca^2+^ equilibrium potential [32]. For the *+/v2J* OHCs this shift resulted in an increased fraction of the transducer channels open at rest. However, the shift was not sufficient to establish a resting transducer conductance at +86 mV for the *v2J/v2J* OHCs. Although the currents of the *v2J/v2J* OHCs had several unusual features, including abnormal directional sensitivity and slow onset kinetics, the disappearance of adaptation at positive potentials cannot be explained by a seal-resistance artefact and identifies the currents as mechano-electrical transducer currents.

### Tip link status in mutants

Electron microscopy was used to assess the status of tip links in *av6J*, *av3J* and *v2J* mice. Difficulties are, however, encountered locating tip links or potential tip link sites in disorganised hair bundles and ambiguities can be caused by the transient lateral links that are present in abundance at the early stages of development. Several types of tissue were therefore examined. Cochlear hair bundles were studied by field emission scanning electron microscopy (FESEM) in the *av6J* mouse at P9, and in both the *av3J* and *v2J* mice at P4. Utricular bundles were examined by transmission electron microscopy (TEM) in the *av6J* and *av3J* mice at P15 and P26, and in the *v2J* mice at P11. In scanning electron micrographs, links were categorized as tip links if they emanated from the tip of a stereocilium in any of the first 3–4 tallest rows and attached to the side of a taller stereocilium in the next row. In immature bundles (i.e., at P3 and P4), such tip links could be directed to either or both of the two nearest taller stereocilia [33], unlike in the mature bundle where tip links primarily are directed to only one taller stereocilium and usually along the excitatory axis [5]. Links categorized as ‘tip links’ therefore do not conform exclusively to the adult form and may represent a combination of transient lateral and/or tip links that are subsequently refined to remove extraneous ones.

**Figure 8:**
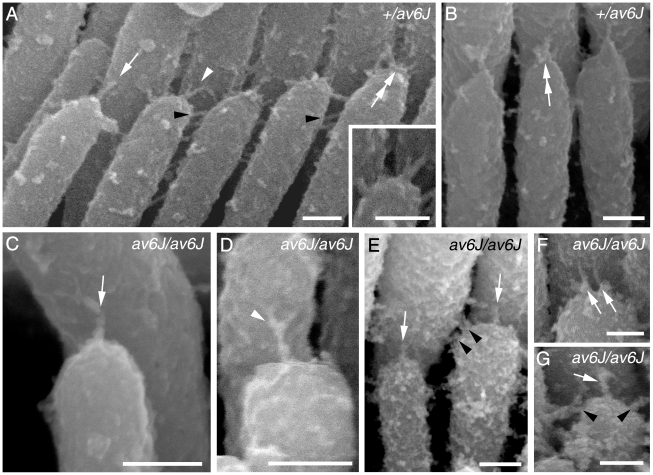
Tip link morphology in *av6J* cochlear hair cells. Tip links from *+/av6J* (A, B) and *av6J/av6J* (C–G) hair cells at P9. (A, B) Heterozygous *+/av6J* control specimens show some single (white arrow in A), forked (white arrowhead and inset in A) and multiple tip links (double white arrows in A and B). Lateral links are also present (black arrowheads in A). (C, D) *av6J/av6J* stereocilia showing a single tip link (white arrow in C) and a forked tip link (white arrowhead in D). (E–G) *av6J/av6J* profiles showing various forms of link occur near the tips. These include single tip-link filaments on adjacent stereocilia (white arrows in E and G), several lateral links (black arrowheads in E and G), and two separate ‘tip links’ from one stereocilium tip (white arrows in F). Scale bars = 100 nm.

Tip-link like links were present in both *+/av6J* (Fig. 8A, B) and *av6J/av6J* cochlear hair cells at P9 (Fig. 8C–G). These tip links included single filaments, forked filaments, broken filaments and multiple filaments (Fig. 8A–E) similar to those described previously in various rodents [5], [33], [34], [35], [36], [37]. Double links were also detected around the tips of some stereocilia (Fig. 8F) and lateral links were seen close to the tips (Fig. 8G). To determine whether the number of these tip-link like links was reduced in *av6J/av6J* mice, 10 randomly selected hair bundles were assessed. The links were assigned to one of four categories: ‘unforked’, ‘forked’, ‘stumps’, or ‘possible’, the latter where a link was morphologically unclear. Expressed as a proportion of possible tip link sites (i.e. tip-side appositions) the homozygote had fewer occupied sites (30%) compared with the heterozygote (46%), fewer unforked links (23% vs 47%) and fewer stumps (5% vs 9%) but more ambiguous links (65% vs 38%). Since most of the hair bundles had some abnormality in row structure, the number of possible sites was smaller in the homozygote than the heterozygote.

**Figure 9:**
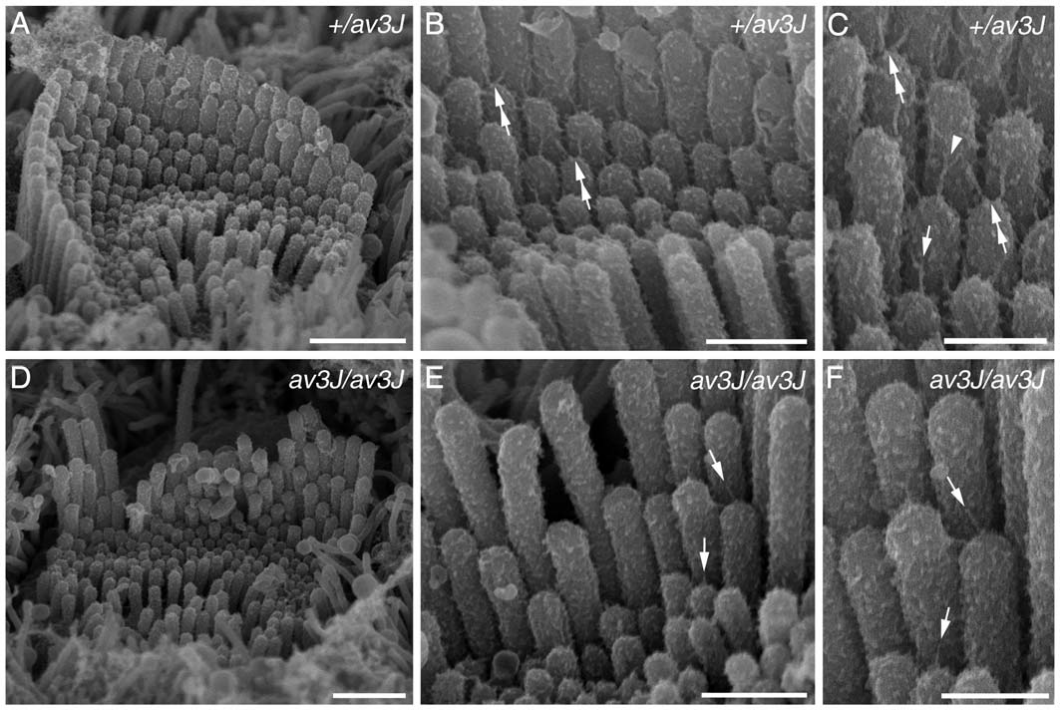
Tip link morphology in *av3J* cochlear hair cells. Links from *+/av3J* (A–C) and *av3J/av3J* (D–F) mice at P3. (A) In the heterozygote, bundles have a regular appearance, similar to those of wild type mice, with stereocilia in precisely aligned rows. Additional short stereocilia, typical of this stage of development, can be seen, all of approximately the same height. Note multiple links can be seen on all the stereocilia. Scale bar = 1 µm. (B) Detail of a bundle from the heterozygote showing links emanating from the tips of the shorter stereocilia and connecting to the sides of the taller stereocilia behind. As is also typical of immature animals, there are sometimes two such links diverging from the same tip onto adjacent taller stereocilia (double white arrows). Scale bar = 500 nm. (C) Detail of the bundle shown in B at higher magnification. Some forked tip links (white arrowhead) are evident and some single filament ones (arrow). Scale bar = 300 nm. (D) Example of a homozygote bundle showing the disrupted bundle structure, with misalignments and gaps in the rows, but also many short stereocilia as in the heterozygote. Scale bar = 1 µm. (E) Detail of a homozygote hair bundle. Although numerous potential tip link sites are evident, and some tip links may be present (arrows), the majority lack tip links or tip-link like filaments. There is also in general fewer links of any kind on these bundles. Scale bar = 500 nm. (F) Detail of an area from E showing several fine tip-link like structures (arrows). Scale bar = 300 nm.

**Figure 10:**
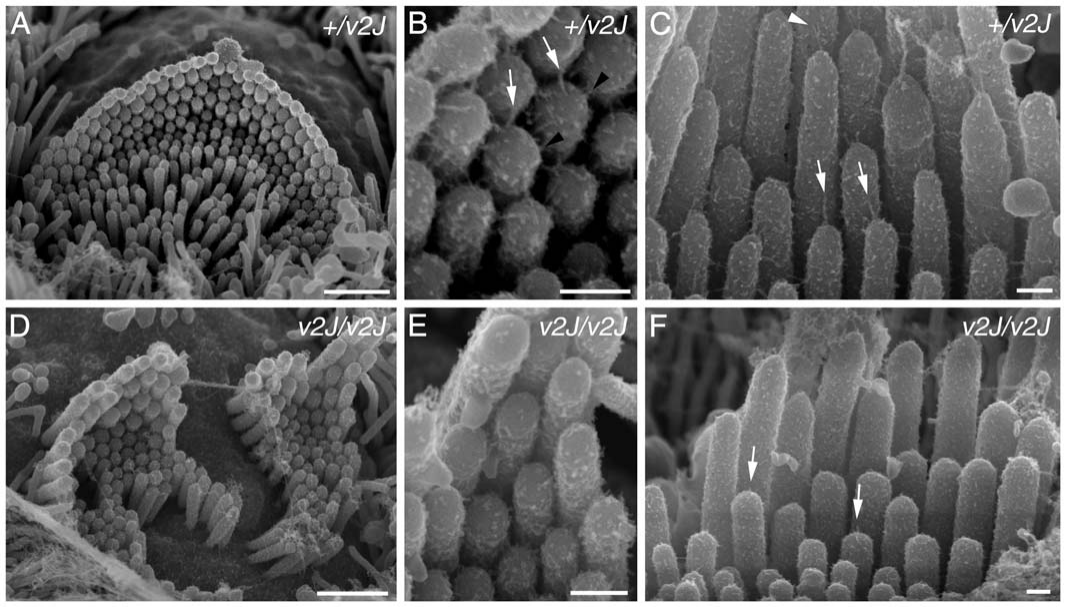
Tip link morphology in *v2J* cochlear hair cells. Links from *+/v2J* (A–C) and *v2J/v2J* (D–F) mice at P4. (A) Heterozygous *+/v2J* OHC row 1 hair bundle. The normal row structure is evident at this stage and the stereocilia are well organised. (B) Detail from the bundle shown in A. Normal tip links (white arrows) and lateral links (black arrowheads) are evident near the tips of the stereocilia. (C) Heterozygous *+/v2J* IHC bundle showing well defined tip links (white arrows), including some that are forked (white arrowhead). (D) Hair bundle from a homozygous *v2J/v2J* apical row 1 OHC. The bundle is distorted by a central split and there is unevenness in the height of the stereocilia within a row, especially the tallest row. (E) Detail of the bundle shown in D. There is little evidence of robust, full-length tip links (compare B and E). (F) IHC from a *v2J/v2J* mouse. Occasional stumpy links are evident near the tips of the stereocilia (white arrows). Scale bars = 1 µm in A and D; 200 nm in B, C, E and F.

Clearly identifiable tip links, including forks, were visible on the OHCs and IHCs from the *+/av3J* (Fig. 9A–C) *+/v2J* mice (Fig. 10A–C) at P4. Because these are early stages, the heterozygotes tended to have spoke-like links typical of immature hair cells [33]. The *av3J/av3J* hair cells had tip-link like links (Fig. 9D–F), whilst the *v2J/v2J* hair cells had no unambiguous full or forked tip links but there were occasional shortened stumps and random filaments at the tips (Fig. 10D–F). Counts from two *av3J/av3J* mice showed 17% and 6% respectively of sites were occupied by some form of link-like structure compared with 67% in the *+/av3J* control. In a *v2J/v2J* mouse the number of occupied sites was 3% compared with 30% in the control. Ranking of the stereocilia was also apparent in both the *av3J/av3J* and the *v2J/v2J* homozygotes although bundle defects were evident.

**Figure 11:**
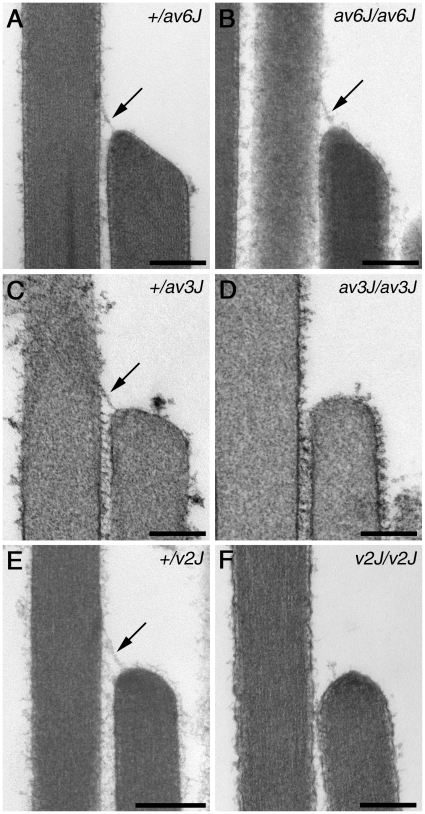
Transmission electron microscopy of vestibular hair bundles in *av6J*, *av3J* and *v2J* mice. Representative TEM images from utricular hair bundles in *+/av6J* (A), *av6J/av6J* (B), *+/av3J* (C), *av3J/av3J* (D), *+/v2J* (E) and *v2J/v2J* (F) mice at P26 (A–B), P15 (C–D) and P11 (E–F). Arrows point to tip links. Scale bars = 200 nm.

Using TEM, tip links could also be documented in mature vestibular hair cells of *av6J/av6J* mice (Fig. 11A, B), but not in *av3J/av3J* (Fig. 11C, D) or *v2J/v2J* (Fig. 11E, F) mice. The number of potential tip link sites that were occupied by tip links (including putative tip links) was evaluated quantitatively. In the *+/av6J* mouse 86% (12/14) of potential sites were occupied by a tip link, whereas only 46% (14/30) were found to have tip links in the *av6J/av6J* mutant. For the *+/av3J* mouse 80% (32/40) sites were occupied whilst 0% (0/43) was occupied in the *av3J/av3J* mutant. For the *+/v2J* mouse 43% (23/53) sites were occupied whereas 4.3% (2/46) were occupied in the *v2J/v2J* mice. The top of the shorter stereocilium at tip-link sites was often found to be wedge-shaped in the *+/av6J* and *av6J/av6J* mice, as well as in the *+/av3J* and the *+/v2J* mice (Fig. 11A–C, E), possibly as a consequence of mechanotransducer channel activity regulating actin polymerisation near the channel. In contrast, the tops of the shorter stereocilia at the unoccupied tip-link sites in the *av3J/av3J* or *v2J/v2J* mice were usually more rounded and dome-shaped (Fig. 11D, F).

## Discussion

The results of this study 1) show that CDH23 and PCDH15 are required for the formation and/or maintenance of a substantial complement of normal tip links, 2) reveal that transducer currents are abnormal in mice with mutations in the genes encoding these proteins, and 3) provide further evidence that ototoxic aminoglycoside antibiotics enter hair cells via their mechanotransducer channels. The study therefore adds to a growing body of data [10], [11], [12], [13], [17] showing that two members of the cadherin superfamily, both of which are products of Usher Type 1 syndrome loci [38], [39], [40], are components of the hair cell's tip link, and that this link is required for normal gating of the hair cell's mechanotransducer channel. Whilst there have been previous reports on the status of transduction in *av3J/av3J* hair cells [41], tip links in *v2J/v2J* cells [35], and the phenotype of *av6J/av6J* hair cells [21], this is the first study in which mice with mutations in the two genes, *Cdh23* and *Pcdh15*, have been compared using a series of tests that specifically probe for both the presence and function of tip links.

SEM and phalloidin-stained preparations of the early postnatal cochlea revealed that hair-bundle structure was severely disrupted in both the *v2J* and the *av3J* mutants, and less so in the *av6J* mutants. The presumed loss-of-function mutations in *Cdh23* (*v2J*) and *Pcdh15* (*av3J*) therefore cause a similar phenotype, and the in-frame deletion of *Pcdh15* (*av6J*) has an effect that is, at least initially, less severe than that observed in either of the presumed nulls. Although a previous study [35] has indicated that tip links are present in the early postnatal cochleae of both *v/v* and *v2J/v2J* mice, we were unable to find links with SEM that could be clearly defined as tip links in the *v2J/v2J* mice. The tip-link like links that are observed in the *v2J/v2J* mice are short and hard to distinguish from the abundant transient links present at these early stages. Tip links were, however, observed in the cochlea of the *av6J/av6J* mice. Whilst their numbers were somewhat reduced, they were clearly present. Likewise, in the relatively mature utricle where the effects of the mutations on hair bundle structure are less severe, we were able to find a number of clear examples of tip links in the *av6J/av6J* mutants with TEM, but not in either the *av3J/av3J* or the *v2J/v2J* mice. The only two links that were considered to be tip links in the utricles of *v2J/v2J* mice were not clear-cut, unambiguous examples of tip links. As in the cochleae, fewer potential tip link sites were occupied by a defined tip link in the *av6J/av6J* mutants than in the heterozygous *+/av6J* controls. These results therefore provide genetic evidence that PCDH15 and CDH23 are both required for the formation and/or maintenance of tip links, and that tip links can form in mutants in which a large part of the 9^th^ cadherin repeat in the ectodomain of PCDH15 is deleted.

Although the deletion in *av6J* cuts out key structural elements from one of the cadherin repeats in PCDH15, several lines of evidence indicate that functional tip links are formed in the *av6J/av6J* mutants. The hair cells load with FM1-43, a dye that is known to enter hair cells via their transducer channels [26], and they are sensitive to aminoglycoside antibiotics, compounds also known to be permeant blockers of the hair cell's transducer channels [30]. Furthermore, transducer currents can be recorded in response to hair bundle movements. If one assumes that the *av6J* deletion generates an entirely non-functional molecule that cannot interact with CDH23, one could argue that PCDH15 is not required for either tip link formation or transduction, despite accumulating evidence (including the data presented above) suggesting otherwise. An alternative and perhaps more parsimonious hypothesis would be that the deleted form of PCDH15 can interact with CDH23 and that tip links do form but are rather unstable. The reduction in the number of tip links observed in the *av6J/av6J* mutants, the reduced staining seen with antibody HL5614 to the PCDH15 ectodomain, the decrease in FM1-43 dye loading and the smaller currents recorded in response to hair bundle displacement all suggest that the latter hypothesis is correct. The behavioural and audiological deficits seen in the young *av6J/av6J* mouse [21] also suggest that the tips links do eventually fail. Whilst it may be possible that an alternative splice variant is used in the *av6J/av6J* mouse that cuts out more of the cadherin repeats in a ‘structurally-feasible’ fashion (i.e., removes one or more entire cadherin repeats including the 9^th^ repeat that is partially encoded by exon 22), the available evidence indicates that 15 of the 17 predicted transmembrane splice variants expressed in the early postnatal cochlea include exon 22 [11], with the two splice variants lacking exon 22 being unlikely to form a protein with a structurally functional ectodomain. Our unpublished data from RT-PCR experiments using primer pairs located either in exons 20 and 23 or in exons 18 and 27 further confirm that spliced forms of *Pcdh15* excluding exon 22 do not exist the early postnatal cochlea.

In response to hair-bundle displacements evoked by a fluid jet, transducer currents could be recorded from the hair cells of the homozygous *av6J/av6J* mouse. The resting transducer conductance was reduced and the evoked currents were, on average, ∼50% lower in amplitude. These findings are, respectively, in accordance with the reduction in FM1-43 dye loading and the decreased number of unambiguous tip links seen in the *av6J/av6J* mouse. In the homozygous *av3J/av3J* and *v2J/v2J* hair cells there was no evidence for a resting current, as also predicted from the FM1-43 experiments in which no dye loading was observed. Quite unexpectedly, however, we found evidence for transduction in both of these presumed null mutants. In the homozygous *av3J/av3J* and *v2J/v2J* OHCs, small transducer currents were observed at negative holding potentials, but in response to force stimuli directed away from the kinocilium, whilst larger transducer currents could be elicited at positive potentials. In contrast to the transducer currents of the homozygous *av6J/av6J* OHCs, which appeared to be a scaled-down version of the currents in their heterozygous *+/av6J* counterparts, the *v2J/v2J* hair bundles required considerable displacement for the transducer channels to open and the polarity of the responses was abnormal, with bundle movements in the negative direction eliciting the largest transducer currents in all but one of the five OHCs tested with step stimuli. Three *v2J/v2J* OHCs tested with force steps responded with transducer currents to both negative (largest) and positive (smaller) stimuli. This suggests a degree of randomization of the directional sensitivity of the transducer channels, as previously reported for *Vlgr1* knockout mice that lack ankle links and have disorganised hair bundles [42].

The transducer currents recorded from *av3J/av3J* and *v2J/v2J* mutants in response to bundle displacements, together with our morphological observations, suggest that some form of transduction may occur in the absence of bona fide tip links (i.e., those formed by the transdimerization of PCDH15 and CDH23 homodimers). Alternative channel gating mechanisms, based on a kinematic analysis of stereociliary shearing and the localisation of a putative mechanotransducer transducer channel to the contact region that lies between stereocilia just below the tip link, have been suggested in the past [43], [44], [45]. Whilst there are some tip-link like links present between the stereocilia at the early stages of development that may mediate transduction events in the *av3J/av3J* and *v2J/v2J* mutants, it is hard to see how these would mediate excitatory responses to deflections that are normally inhibitory (i.e., away from the kinocilium). A gating mechanism for the mechanotransducer channels that lacks the directional sensitivity provided by an oblique tip link would be an alternative hypothesis, such as that proposed for inner (but not outer) hair cells in MYO15A mutant mice [46]. One possibility for the *av3J* and *v2J* mutants described above is that loss of normal tip links results in the channels being mislocalised to sites where they could be gated by the lateral links. This would imply that a residual gating element is present that enables the channels to be activated by stresses imposed on the membrane even in the absence of the normal link.

Finally it can be noted that there are additional similarities between the hair cells of the *v2J/v2J* mouse defective in CDH23 and the *av3J/av3J* mouse defective in PCDH15 described here and those of the shaker1 mouse that is defective in MYO7A [28]. The hair cells from all three mutants have all channels closed at rest and fail to load with FM1-43. The hyperadaptation and slow onset kinetics of the transducer currents of the *v2J/v2J* mutant OHCs we identified with step stimuli are similar to the currents we observed previously in shaker 1 mutant mice [28]. Like in shaker 1 mutants, the hair cells of the *av3J/av3J* mutant mice do not accumulate [^3^H]-gentamicin and are insensitive to aminoglycosides. The cytoplasmic tail of MYO7A interacts directly with CDH23 [47], and CDH23 in turn is known to interact with PCDH15 [13]. Our results are therefore entirely consistent with the concept that the transducer channels are the route of entry for aminoglycosides into hair cells, and that MYO7A can, either directly or indirectly, modulate the open probability of these channels.

To conclude, our results together with those of previous studies provide genetic evidence for the concerted involvement of PCDH15, CDH23 and MYO7A in the formation and regulation of the tip-link complex in mechanosensory hair cells. They also raise the intriguing possibility that the mechanotransducer channels may, under certain circumstances, be gated via mechanisms that do not involve the classic, oblique tip link.

## Materials and Methods

### Mice

The use of mice at Case Western Reserve University (CWRU) was approved under protocol number 2010-0074 (entitled ‘Characterization of mouse models of deafness’) by the Institutional Animal Care and Use Committee (IACUC). The main objective of protocol 2010-0074 is to use various tools and techniques to understand the molecular basis of hearing and deafness using mouse models. At the University of Sussex, all animal procedures were performed under a UK Home Office Project licence (PPL70/6721 entitled Molecular, Cellular and Physiological Basis of Hearing and Deafness) and with the approval of The University of Sussex Ethical Review Committee.

To study changes associated with mutations in *Pcdh15*, two alleles of Ames waltzer (*av*) were used: *Pcdh15^av6J^* [21] and *Pcdh15^av3J^* [48]. Most of the data from the *av* mice was compared to that obtained from hair cells from mice with a mutation in *Cdh23*, the waltzer *v2J* allele. Mice homozygous for the *av6J* allele (*av6J/av6J*) were crossed to mice heterozygous for av*6J* allele (*+/av6J*) and offspring were genotyped using a PCR-based approach [21]. A similar breeding scheme was used to obtain mice heterozygous (*+/av3J*) or homozygous (*av3J/av3J*) for the *av3J* allele of *Pcdh15*, and mice that were heterozygous (*+/v2J*) or homozygous (*v2J/v2J*) for the *v2J* allele of *Cdh23*. Offspring from the *av3J* and *av6J* lines were genotyped as described previously by Pawlowski et al and Zheng et al respectively [21], [49]. Offspring from the *v2J* line were genotyped using a scheme that takes advantage of the point mutation that results in the loss of HpyCH4 III restriction site in the target sequence. Genomic DNA (∼500 ng/reaction) was amplified under standard reaction conditions using Platinum Taq DNA polymerase (Invitrogen, CA). The reaction mix was incubated at 94°C for 120 s followed by 35 cycles of 94°C for 30 s, 55°C for 20 seconds and 72°C for 30 s, 72°C for 45 s. Primer pairs and product size are as follows: Primer KA1063 – 5′CGA GAC CAA GAC CAG CTA CC 3′ and KA1064 – 5′ACC AGA CTG ACT GGC TTT CA, 412 bp. The PCR product was digested with HpyCH4 III (NEB, MA) according to manufacturer's protocol followed by heat inactivation at 80°C for 15 minutes. The digested sample was resolved on a 3% agarose gel. Three bands (284, 88 and 40 bp) were observed with wild-type allele and two bands (372 and 40 bp) were observed for the *v2J* allele.

### Cochlear culture preparation

Cochlear organ cultures were prepared from *av3J*, *av6J* and *v2J* mice at 0–3 days of age as described previously [23] and maintained on collagen-coated glass coverslips in Maximow side assemblies at 37°C in a medium containing 93% DMEM/F12, 7% foetal bovine serum and 10 µg/ml ampicillin for 24 h before use.

### FM1-43 labelling

FM1-43 dye loading experiments were performed as described previously [26]. In brief, coverslips with adherent cochlear cultures were washed once with HEPES Buffered (10 mM, pH 7.2) Hanks' Balanced Salt Solution (HBHBSS), dipped for 10 seconds in HBHBSS containing 3 µM FM1-43, and washed immediately three times in a large volume of HBHBSS (10 seconds for each wash). The coverslips were then placed in a glass-bottomed Perspex chamber containing 0.5 ml HBHBSS and viewed with an upright microscope equipped with epifluorescence optics and FITC filters (excitation 488 nm, emission 520 nm) using a 63× water immersion or a 40× dry objective. Images were captured at fixed time points after dye application using a 12-bit cooled CCD camera (SPOT-JNR). FM1-43 levels were quantified for *+/av6J* and *av6J/av6J* hair cells (see Fig. 4 A–D). Images were imported into Adobe Photoshop CS3 and grey levels were measured in the cytoplasm between the cuticular plate and hair-cell nuclei for 23 hair cells for each genotype, for both the apical and basal coils, using a 400 pixel (20×20) region of interest. Statistical significance was assessed using one-way ANOVA followed by the Tukey test, with *P*<0.05 as the criterion. The number of cochlear cultures used, each of which contained two apical-coil and two basal-coil explants, was: *+/av3J* (n = 4), *+/av6J* (n = 11), *av3J/av3J* (n = 4), *av6J/av6J* (n = 6). Similarly, apical- and basal-coil explants from *+/v2J* (n = 5) and *v2J/v2J* (n = 6) were cultured for FM1-43 labeling.

### [^3^H]-gentamicin labelling

[^3^H]-gentamicin labelling was performed as described previously [29]. Briefly, coverslips with adherent cochlear cultures were removed from Maximow slide assemblies, placed in 35 mm diameter plastic culture dishes, washed three times with HBHBSS, and incubated in HBHBSS containing 0.1 mM [^3^H]-gentamicin (Amersham, UK) for 2 h at 37°C. Cultures were then placed on ice, washed three times with 4 ml of ice cold HBHBSS over a 10 minute period and fixed in cold 2.5% glutaraldehyde buffered with 0.1 M sodium cacodylate, pH 7.2 for 1 h. Following glutaraldehyde fixation, cultures were washed thrice in 0.1 M cacodylate buffer, fixed for 1 h with 1% osmium tetroxide, washed in buffer, dehydrated with ethanol and embedded in Epon 812 resin (TAAB, Berks, UK). One-micrometer thick sections of the apical and basal-coil cochlear cultures were cut from the plastic blocks with glass knives, mounted on glass slides and either stained with Toluidine blue or coated with Ilford L4 Nuclear Research Emulsion (Ilford Imaging UK Limited, Mobberley, Cheshire) for autoradiography. Emulsion-coated slides were exposed in light-tight boxes in the presence of dessicant at 4°C for 1 to 3 weeks, and developed in Ilford Phenisol (diluted 1+4 with H_2_O) for 4 min at 18°C. Developed autoradiographs were washed, fixed and dried, mounted under glass coverslips in Histomount, and photographed with an Axioplan 2 light microscope using phase contrast optics.

### Aminoglycoside treatment

To test for sensitivity to aminoglycoside exposure, coverslips with adherent cochlear cultures were placed in 35 mm plastic petri dishes, washed once with 3 ml of HBHBSS and then incubated in 3 ml of HBHBSS or 3 ml of HBHBS containing 1 mM neomycin or 1 mM gentamicin at 37°C for 2–4 hours. Following a brief wash, cultures were fixed as described above for grain density autoradiography. Effects of aminoglycosides were evaluated by light microscopy using 1 micron-thick, Toluidine blue-stained sections of basal-coil cultures.

### Recording mechanotransducer currents

Mechano-electrical transducer currents from apical-coil outer hair cells were studied in organotypic cochlear cultures of *av3J* mice (P2 plus two days in vitro) and *v2J* mice (P1 plus 2 or P0 plus 3 days in vitro), and from the cochleae of *av6J* mice that were acutely dissected at P4 and P5. All experiments were at room temperature (22–25°C). Extracellular solution was continuously bath-applied at a rate of 6 ml/h. It contained (in mM): 135 NaCl, 5.8 KCl, 1.3 CaCl_2_, 0.9 MgCl_2_, 0.7 NaH_2_PO_2_, 2 Na-pyruvate, 5.6 D-glucose, 10 HEPES, with amino acids and vitamins for Eagle's minimum essential medium (MEM) added from concentrates (Invitrogen, UK). The pH was adjusted to 7.5 and the osmolality was about 308 mmol kg^−1^. The organs of Corti were observed with an upright microscope (Zeiss ACM or Leica DMLFSA, Germany) with Nomarski optics.

Transducer currents were elicited using fluid-jet stimulation (45 Hz sinewaves or steps filtered at 1.0 or 3.0 kHz, 8-pole Bessel) and recorded under whole-cell voltage clamp (HEKA EPC8, Germany or Cairn Optopatch, UK) as previously described [24], [50]. The maximum driver voltage to the fluid jet was set at levels (35 to 40 V) that elicited large, near-saturating transducer currents in control cells. Patch pipettes (resistance in the bath 2–3 MΩ) were pulled from soda glass capillaries and coated with wax. Intracellular solutions contained (in mM): 147 CsCl, 2.5 MgCl_2_, 1 EGTA-CsOH, 2.5 Na_2_ATP, 5 HEPES; pH adjusted to 7.3 with 1 M CsOH. Data were filtered at 2.5–3.0 kHz, sampled at 5 kHz and stored on computer for off-line analysis using Origin software. Membrane capacitance (C_m_) was 5.9±0.3 pF and series resistance after electronic compensation of up to 70% (R_s_) was 2.5±0.3 MΩ, resulting in voltage-clamp time constants of 15±2 µs (n = 21). Membrane potentials were corrected for a −4 mV liquid junction potential between pipette and bath solutions. No correction was made for the voltage drop across this series resistance, which was at most 2 mV at extreme potentials. Voltage clamp protocols are referred to a holding potential of −84 mV. Mean values are quoted ± s.e.m. in text and figures.

### Immunofluorescence microscopy

Cochleae or cochlear cultures were fixed by immersion in 3.7% formaldehyde in 0.1 M sodium phosphate buffer for 1 hour at RT, and washed 3 times in PBS. After removal of the cartilaginous capsule and the lateral wall, cochlear coils were preblocked and permeabilised in TBS containing 10% heat inactivated horse serum and 0.1% TX-100 for 1 hour, and incubated overnight in the same solution containing a 1∶100 dilution of each of the following affinity purified rabbit antibodies directed against PCDH15 (a kind gift from Dr. Tom Friedman, NIDCD): PB303, 464-2B and PB375 recognising the cytoplasmic domains of the CD1, CD2 and CD3 isoforms classes of PCDH15 respectively, and HL5614 directed against an N-terminal ectodomain sequence common to all classes. Antibody HL5614 was used in the presence of 2 mM EDTA. Samples were washed 3× with TBS, and stained with FITC conjugated swine anti-rabbit Ig (1∶100 dilution) and rhodamine conjugated phalloidin (1∶1000 dilution) for 2 h, washed in TBS, mounted in Vectashield and viewed with a Zeiss LSM 510 confocal microscope using a 100× oil immersion lens NA1.4. To examine hair bundle morphology in *av6J* mice, cochlear coils were stained only with Texas Red conjugated phalloidin.

### Transmission electron microscopy

Utricles from *av3J* mice at P15 and P26 and *av6J* mice at P15 were dissected in HBHBSS and fixed immediately in glutaraldehyde followed by osmium tetroxide and embedded in resin as described above for grain density autoradiography. For *v2J* mice the utricular maculae were dissected in 70% ethanol from labyrinths that had been immersion fixed in glutaraldehyde followed by osmium. Thin (∼90 nm thick) sections were cut with a diamond knife, mounted on copper grids, double stained with uranyl acetate and lead citrate [51]. Sections were viewed in a Hitachi 7100 transmission electron microscope and images were captured with a Gatan Ultrascan 1000 CCD camera. Sections from the utricles of 2 *+/av6J*, 4 *av6J/av6J*, 6 *+/av3J*, 7 *av3J/av3J*, 4 *+/v2J* and 2 *v2J/v2J* mice were examined by transmission electron microscopy.

### Scanning electron microscopy

Cochleae from heterozygous and homozygous *av6J* mice at P9, *v2J* at P4 and *av3J* mice at P3 were fixed by intra-labyrinthine perfusion with 2.5% glutaraldehyde in 0.1 M sodium cacodylate buffer containing 2 mM CaCl_2_ (pH 7.4) and then immersed in the fixative for 2 h. They were stored in 1/10^th^ concentration fixative (0.25% glutaraldehyde) diluted with buffer at 4°C until further processing, subsequently washed in buffer, dissected to reveal the organ of Corti, and post-fixed with cacodylate-buffered 1% OsO_4_ for 1 h. After washing in cacodylate buffer, samples were impregnated with osmium using the osmium-thiocarbohydride (OTOTO) method; for details see [52]. After OTOTO they were dehydrated through a series of increasing ethanol concentrations up to 100% ethanol dried over molecular sieve, and critical point dried from liquid CO_2_ using a Polaron critical point dryer. Cochleae were affixed to specimen stubs using silver conducting paint (Agar Scientific, Stansted, UK) and examined in a Hitachi S4500 field emission SEM operated at 5 kV. Specimens from the cochleae of 4 *+/av6J*, 2 *av6J/av6J*, 2 *+/v2J*, 2 *v2J/v2J*, 2 +/*av3J* and 2 *av3J/av3J* mice were examined by scanning electron microscopy.
